# Growth inhibition of different human colorectal cancer xenografts after a single intravenous injection of oncolytic vaccinia virus GLV-1h68

**DOI:** 10.1186/1479-5876-11-79

**Published:** 2013-03-26

**Authors:** Klaas Ehrig, Mehmet O Kilinc, Nanhai G Chen, Jochen Stritzker, Lisa Buckel, Qian Zhang, Aladar A Szalay

**Affiliations:** 1 Department of Biochemistry, University of Wuerzburg, Wuerzburg, Germany; 2 Genelux Corporation, San Diego Science Center, San Diego, California, USA; 3 Department of Radiation Medicine and Applied Sciences, Rebecca & John Moores Comprehensive Cancer Center, University of California, San Diego, California, USA; 4 Rudolf Virchow Center for Experimental Biomedicine, University of Wuerzburg, Wuerzburg, Germany; 5 Institute for Molecular Infection Biology, University of Wuerzburg, Wuerzburg, Germany

**Keywords:** Cancer, Vaccinia virus, Colorectal, Oncolytic virotherapy, Metastasis

## Abstract

**Background:**

Despite availability of efficient treatment regimens for early stage colorectal cancer, treatment regimens for late stage colorectal cancer are generally not effective and thus need improvement. Oncolytic virotherapy using replication-competent vaccinia virus (VACV) strains is a promising new strategy for therapy of a variety of human cancers.

**Methods:**

Oncolytic efficacy of replication-competent vaccinia virus GLV-1h68 was analyzed in both, cell cultures and subcutaneous xenograft tumor models.

**Results:**

In this study we demonstrated for the first time that the replication-competent recombinant VACV GLV-1h68 efficiently infected, replicated in, and subsequently lysed various human colorectal cancer lines (Colo 205, HCT-15, HCT-116, HT-29, and SW-620) derived from patients at all four stages of disease. Additionally, in tumor xenograft models in athymic nude mice, a single injection of intravenously administered GLV-1h68 significantly inhibited tumor growth of two different human colorectal cell line tumors (Duke’s type A-stage HCT-116 and Duke’s type C-stage SW-620), significantly improving survival compared to untreated mice. Expression of the viral marker gene *ruc-gfp* allowed for real-time analysis of the virus infection in cell cultures and in mice. GLV-1h68 treatment was well-tolerated in all animals and viral replication was confined to the tumor. GLV-1h68 treatment elicited a significant up-regulation of murine immune-related antigens like IFN-γ, IP-10, MCP-1, MCP-3, MCP-5, RANTES and TNF-γ and a greater infiltration of macrophages and NK cells in tumors as compared to untreated controls.

**Conclusion:**

The anti-tumor activity observed against colorectal cancer cells in these studies was a result of direct viral oncolysis by GLV-1h68 and inflammation-mediated innate immune responses. The therapeutic effects occurred in tumors regardless of the stage of disease from which the cells were derived. Thus, the recombinant vaccinia virus GLV-1h68 has the potential to treat colorectal cancers independently of the stage of progression.

## Background

According to the American Cancer Society, cancer is the second leading cause of death, with an estimated total of 577,190 deaths worldwide in 2012. Colorectal cancer is the third-most diagnosed cancer and the fourth leading cause of cancer death in both sexes. In 2012 in the United States alone, there were estimated to be 103,170 colorectal cancer-related deaths [1]. While standard treatments of colorectal cancer can be up to 90% effective for early stage disease, the 40-60% of patients with recurring or late stage disease have few effective treatment options.

Colorectal cancer begins as a benign adenomatous polyp and eventually develops into a malignant and, if untreated, metastatic cancer over the course of several years. The most frequent mutations associated with colorectal cancer are in genes of the Wnt signaling pathway, the *p53* and the *TGF-β* genes. Colon cancers identified at early (I & II) stages of the disease are highly treatable and can oftentimes be cured with surgical resection being the standard therapy [2]. Adenocarcinomas account for more than 95% of reported cases, making it the most common colorectal cancer cell type. Surgical excision provides cure rates of 90% in stage I and 75% in stage II, and postsurgical combination therapy with 5-Fluorouracil-based chemotherapeutic agents increases the survival rate in stage III disease from 40% to 60% [3]. However, cases of surgical excision and chemotherapy still show recurrence rates between 40-60% in the first three years, with similarly high recurrence rates at later stages of the disease, likely caused by chemoresistant cancer-initiating cells [4,5]. Metastatic stage IV disease is incurable and treatment becomes palliative. Newer and more efficient treatment regimens are needed to reduce the still considerably high treatment failure rates, especially in recurring cases or the late stages of colorectal cancer disease.

Among new therapeutic strategies, oncolytic virotherapy has been explored recently for its target specificity and relative safety in patients. Oncolytic viruses can efficiently infect and kill tumor cells. These effects have been observed for a number of viruses, including adenovirus, West Nile virus, herpes simplex virus, measles virus, Newcastle disease virus, and vaccinia virus [6,7]. While studies suggest that some of these viruses may have potential for oncolytic virotherapy of colorectal cancer [8-10], none yet have been approved for treatment.

One of the best studied oncolytic viruses is vaccinia virus. The antitumor effects of vaccinia virus are mediated directly by viral infection, replication and lysis of cancer cells and indirectly by inducing antivascular effects [11] as well as stimulation of the host immune response [12]. Moreover, a significant advantage of vaccinia virus is its long history of safe administration in humans as a smallpox vaccine.

We reported previously the tumor selectivity and anti-tumoral efficacy of the replication-competent recombinant vaccinia virus GLV-1h68 in different canine [13,14] as well as human tumor xenograft models like breast cancer [15], anaplastic thyroid carcinoma [16,17], malignant pleural mesothelioma [18], pancreatic tumor [19], prostate carcinoma [20], squamous cell carcinoma [21], sarcomas [22], and hepatocellular carcinoma [23]. In addition, we showed functionality of second-generation recombinant vaccinia viruses armed with the human norepinephrine transporter [24] and the human sodium iodide symporter gene [25] for PET imaging, or single-chain antibody GLAF-1 targeting VEGF and tumor vascularization *in vivo*[26]. We also demonstrated preferential replication in glioblastoma cells and higher efficacy in treating gliomas in combination with radiation therapy [27]. Recently, the first clinical Phase I trial of GLV-1 h68 was completed [28] and three additional Phase I trials are currently ongoing (http://www.clinicaltrials.gov, 2012, keyword: Genelux).

Here, we report for the first time the efficacy of oncolytic vaccinia virus GLV-1h68 to infect, replicate in and lyse various colorectal cancer cells in culture and in tumor xenograft models, and we evaluate these effects on cells derived from patients at different stages of progression of disease in culture and tumor xenograft models.

## Methods

### Cell culture

African green monkey fibroblasts (CV-1) and human colorectal adenocarcinoma cells [HT-29 (Duke’s type B)] were obtained from ATCC (ATCC-No. CCL-70 & HTB-38). Human colon adenocarcinoma cells [Colo 205 (Duke’s type D), HCT-15 & SW-620 (Duke’s type C)] and human colon carcinoma cells [HCT-116 (Duke’s type A)] were obtained from NIH as part of the NCI-60 collection. CV-1 cells were cultured in DMEM (Cellgro) supplemented with 1× antibiotic-antimycotic solution [100 U/mL penicillin G, 250 ng/mL amphotericin B, and 100 U/mL streptomycin) (Cellgro) and 10% fetal bovine serum (FBS)(Cellgro)]. HCT-15, HCT-116, HT-29 and SW-620 cells were cultured in RPMI 1640 (Cellgro) supplemented with 1× antibiotic-antimycotic solution (100 U/mL penicillin G, 250 ng/mL amphotericin B, and 100 U/mL streptomycin) and 10% FBS. Colo 205 cells were cultured in RPMI 1640 supplemented with 1× antibiotic-antimycotic solution [100 U/mL penicillin G, 250 ng/mL amphotericin B, and 100 U/mL streptomycin), 1.5 g/L sodium bicarbonate (Cellgro), 4.5 g/L glucose (Cellgro), 10 mM HEPES (Cellgro), 1.0 mM sodium pyruvate (Cellgro) and 10% FBS]. Cells were cultured at 37°C under 5% CO_2_.

### Virus

The recombinant vaccinia virus GLV-1h68 used in this study is a genetically stable, attenuated virus with oncolytic activity and was derived and purified from vaccinia virus strain LIVP as described earlier [15]. Briefly, three expression cassettes encoding for *Renilla* luciferase *Aequoria* GFP fusion protein, β-galactosidase and β-glucoronidase were inserted into the *F14.5 L*, *J2R* and *A56R* loci, respectively, of the viral genome of the LIVP strain.

### Viral proliferation assay

Standard plaque assays were performed to quantify viral replication following infection of different colorectal cancer lines with GLV-1h68. Colorectal cancer (CRC) cells were infected with GLV-1h68 at a multiplicity of infection (MOI) of 0.01, 0.1 or 10 for 1 h at 37°C and 5% CO_2_. Afterwards, the infection medium (RPMI 1640 supplemented with 2% FBS) was removed and cells were cultured in fresh growth medium (RPMI 1640 supplemented with 10% FBS). Cells were then harvested mechanically in triplicates after 6, 24, 48, 72 and 96 hours post infection (hpi). Following three freeze-thaw cycles, serial dilutions of the samples were titrated in triplicates on confluent layers of CV-1 cells in 24-well plates. After incubation for 48 h at 37°C and 5% CO_2_, plaques were stained with cystal violet solution (crystal violet (Sigma) in 5% (w/v) ethanol (Sigma) and 30% (w/v) formaldehyde (Fisher)).

### Infection of cell cultures

Colorectal cancer cells and CV-1 cells were seeded into 24-well plates to achieve 95% confluence the next day. After 24 h in culture, cells were infected with GLV-1h68 at MOIs of 1.0 and 0.1 in infection medium. Cells were incubated for 1 h at 37°C, after which the infection medium was removed, and cells were cultured in fresh growth medium.

### Cell viability assay

The amount of viable cells after infection with GLV-1h68 was measured using 3-(4,5-Dimethylthiazol-2-yl)-2,5-diphenyltetrazoliumbromid (MTT) (Sigma). Twenty-four, 48 and 72 hpi, the medium was replaced with 0.5 mL sterile MTT solution at a concentration of 2.5 mg/mL MTT dissolved in RPMI 1640 without phenol red and incubated for 2 hours at 37°C in a 5% CO_2_ atmosphere. After removal of the MTT solution, the color reaction was stopped by adding 1 N HCl diluted in isopropanol. The optical density was then measured at a wavelength of 570 nm using a SpectraMax microplate reader (Molecular Devices). Uninfected cells were used as a reference and considered as 100% viable.

### Cell proliferation assay

The proliferation rate of viable HCT-15 and HCT-116 was measured using 2,3-bis-(2-methoxy-4-nitro-5-sulfophenyl)-2H-tetrazolium-5-carboxanilide (XTT) (Roche). HCT-15 and HCT-116 cells were seeded at a concentration of 1×10^3^ cells/well in 96-well plates and proliferation was measured after 24, 48, 72, 96 and 120 hours. Substrate was added according to manufacturer’s instructions and cells were incubated for four hours at 37°C. Optical density was measured at wavelengths of 450/700 nm using a SpectraMax microplate reader (Molecular Devices).

### Fluorescence microscopy

An inverted microscope (Olympus IX71) was used to capture images with a MicroFire® (Olympus) digital CCD camera. Brightfield and fluorescence images were taken 24, 48 and 72 hpi to follow the course of infection and pseudocolored using the open source GIMP2.6 software.

### Flow cytometry analysis

Infected cells were analyzed 24, 48 and 72 hpi. Cells were harvested by Trypsin-EDTA (Cellgro) and resuspended in PBS (Cellgro). For discrimination between viable and dead cells, CRC cells were stained using 5 μL propidium iodide solution (1 mg/mL; Molecular Probes) per 0.5 mL cell suspension for 20 min at 37°C. A total of 7.5×10^4^ cells per sample were then measured for GFP and propidium iodide signals using a Cell Lab Quanta™ SC flow cytometer (Beckman Coulter) and analyzed using Quanta Analysis software (Beckman Coulter).

### Subcutaneous HCT-116 and SW-620 xenografts

Mice were cared for in accordance with approved protocols by the Institutional Animal Care and Use Committee of Explora Biolabs (San Diego Science Center, protocol number EB11-025). Five- to six-week old male Hsd:athymic Nude-*Foxn1*^*nu*^ mice (Harlan) were implanted subcutaneously (s.c.) with 5×10^6^ HCT-116 cells or SW-620 cells (in 100 μL PBS) into the right hind leg. Treatment started when tumors reached a volume of 200–300 mm^3^. GLV-1h68 was administered systemically by intravenous (i.v.) injection into the lateral tail vein of 5×10^6^ plaque-forming units (pfu) in 100 μL PBS at day 0. Control animals were injected i.v. with 100 μL PBS only. Tumor growth was measured using a digital caliper and tumor volume was calculated as 0.5 × (height-5) × width × length (mm^3^). Average tumor volume (ATV) was used to monitor therapeutic efficacy. Net body weight was calculated from the measured body weight (body weight – tumor volume/1000 mm^3^) to exclude tumor mass. Mice were sacrificed when the body weight dropped by one third of their original body weight or the tumor volume exceeded 4000 mm^3^. The experiment was terminated 42 days post injection (dpi).

### Vaccinia viral titers in tumor xenografts and body organs

Tumors and body organs (spleen, kidney, liver, testes, lungs) of five virus-treated animals were excised at 7 and 14 days post injection and placed in two volumes (w/v) of homogenization buffer (50 mM Tris–HCl (pH 7.4) (Fisher), 2 mM EDTA (pH 7.4) (Sigma)) supplemented with Complete Protease Inhibitor Cocktail (Roche Diagnostics). Tumors were then homogenized using a MagNA Lyser (Roche Diagnostics) at a speed of 6,000 for 30 s (three times). Following three subsequent freeze-thaw cycles (liquid N_2_/37°C water bath), supernatants were collected by centrifugation (6,000 rpm, 5 min, 4°C). Viral titers were measured by standard plaque assays on CV-1 cells.

### Preparation of tumor lysates for mouse immune-related protein profiling

Tumor lysates were prepared, at 21 days post injection, from three mice of each treatment group. Tumors were excised surgically, weighed and resuspended in 9 volumes (w/v) lysis buffer [50 mM Tris–HCl (pH 7.4), 2 mM EDTA (pH 7.4), 2 mM PMSF and Complete Mini protease inhibitors (Roche)] and homogenized using a MagNA Lyser (Roche Diagnostics) at a speed of 6,000 for 30 s (three times). Supernatants were collected by centrifugation (6,000 rpm, 5 min, 4°C) and analyzed for mouse immune-related protein antigen profiling by Multi-Analyte Profiles (mouse MAPs; Rules Based Medicine) using antibody linked beads. Results were normalized based by the total protein concentration of each sample and presented as mean antigen amount [n=3] per mg total protein. Total protein concentrations were determined using the DC Protein Assay Kit (BioRad) according to the manufacturer’s instructions.

### Preparation of single cell suspensions and fluorescence-activated cell sorting

Single cell suspensions of tumors were prepared at 21 days post virus injection from four untreated and five treated animals. Tumors were surgically excised, weighed, and minced into small (1–2 mm^3^) pieces with a scalpel, and immersed in 10 mL of digestion mixture [5% FBS in RPMI 1640, 0.5 mg/mL collagenase D (Roche), 0.2 mg/mL hyaluronidase, type V (Sigma), and 0.02 mg/mL DNase I (Sigma)] per 0.25 g of tumor tissue. The suspension was incubated with agitation at 37°C for 45 min. The suspension was then filtered sequentially through 70- and 40-μm cell strainers (BD Falcon) and washed with 5% FBS in RPMI 1640. Red blood cells (RBCs) were lysed by brief incubation with an ammonium chloride-based lysis buffer (BD Biosciences) and cell debris/dead cells were removed by centrifugation. The single cell suspensions obtained were labeled with F4/80 (BD Biosciences) / CXCR4 (BD Biosciences) or CD19 (BD Biosciences) / DX5 (BD Biosciences) antibodies for macrophage or NK cell identification, respectively, and analyzed on a two-laser FACSCanto (BD Biosciences) fluorescence cell sorter. CountBright™ counting beads (Invitrogen) were used according to the manufacturer’s instructions to quantify total cell numbers in the samples.

### Histological analysis of tumors

Tumors were surgically excised and snap-frozen in liquid N_2_, followed by fixation in 4% paraformaldehyde (EMS)/PBS at pH 7.4 for 16 h at 4°C. Tissues were washed in PBS and embedded in 5% low melt agarose (Fisher). Tissues were cut using a VT1200S (Leica) vibratom into 100 μm sections and subsequently permeabilized in 0.2% Triton-X 100 (Fisher), 5% FBS in PBS. GFP expression was used as an indicator for viral distribution within the tumor tissue. Phalloidin-TRITC (Sigma) was used to label actin. The fluorescent-labeled preparations were examined using a Leica MZ 16 FA Stereo-Fluorescence microscope equipped with a FireWire DFC/IC monochrome CCD camera (Leica). Digital images were processed with GIMP2 (Freeware) and merged to yield pseudocolored images.

### Expression of the virus-encoded marker gene GFP

GFP expression within tumors was detected under blue light using a stereo fluorescence macroimaging system (Lightools Research). GFP expression was scored using a four point system: 0) no GFP signal, 1) one spot, 2) two or three local spots, 3) diffuse signal from half the tumor, 4) strong signal from the whole tumor.

### Statistical analysis

Statistical analyses were performed with SPSS, version 11 (SPSS, Inc.). Comparisons of treatment groups were made by ANOVA, and the differences between the groups were analyzed with a least significant difference (LSD) test when the ANOVA showed an overall significance. Values of P less than 0.05 were considered significant.

## Results

### Replication and cytotoxicity of GLV-1h68 in human colorectal cancer cell lines

**Figure 1:**
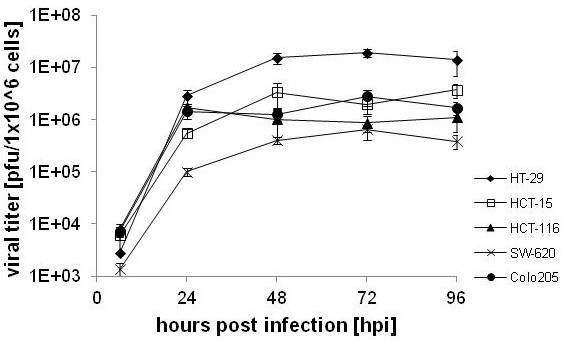
**Analysis of viral titers after infection of five different CRC lines with GLV-1h68 in culture.** Cells were infected at an MOI of 0.1 and samples were collected at various times after infection. Titers were averaged from triplicates and normalized to pfu/1x10^6^ cells. Averages plus standard deviations are plotted.

The replication of recombinant vaccinia virus GLV-1h68 was analyzed in different human colorectal cancer cells in culture (Colo 205, HCT-15, HCT-116, HT-29 and SW-620). These cells represent the common colorectal tumor cell types, human colon adenocarcinoma (HCT-15, HCT-116, HT-29 and SW-620) and human colon carcinoma (Colo 205), and were obtained from patients at all four stages of disease (Duke’s type A through D). Duke’s classification is a widely applied classification system for colorectal cancer, the predecessor of the current TNM staging system. HCT-116 (Duke’s type A), HT-29 (Duke’s type B) and HCT-15 cells (Duke’s type C)are derived from primary tumors whereas SW-620 cells (Duke’s type C) were derived from the lymph nodes and Colo 205 cells (Duke’s type D) from the ascites. While GLV-1h68 replicated in all five cell lines (Figure 1) the replication efficiency between the infected cell lines varied. Higher titers after 24 hpi were observed for HT-29 (2.9×10^6^ ± 8.5×10^5^ pfu/10^6^ cells), HCT-116 (1.7×10^6^ ± 2.6×10^5^ pfu/10^6^ cells) and Colo205 cells (1.4×10^6^ ± 3.8×10^5^ pfu/10^6^ cells) and somewhat lower titers were observed for HCT-15 (5.5×10^5^ ± 1.1×10^4^ pfu/10^6^ cells) and SW-620 cells (9.9×10^4^ ± 1.6×10^4^ pfu/10^6^ cells). Titers in the tested cell lines reached a maximum after 48 to 72 hours post infection (1.91×10^7^ pfu/10^6^ cells in infected HT-29 cells and 6.58×10^5^ pfu/10^6^ cells in SW-620), except for HCT-116 and Colo 205, which peaked 24 hours post infection.

**Figure 2:**
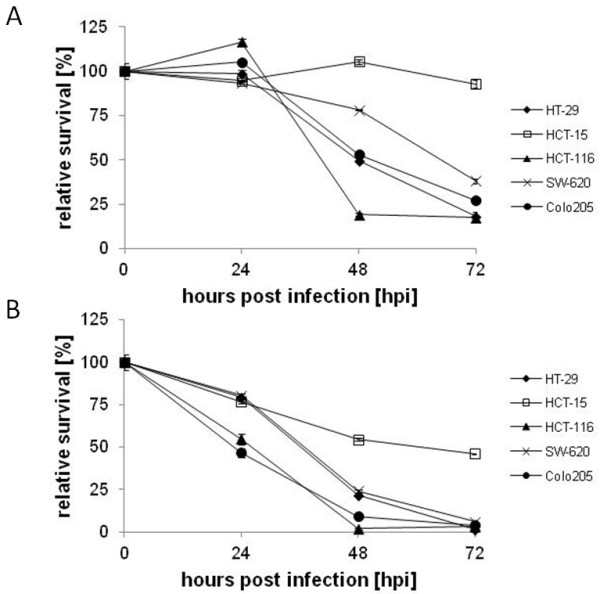
**Cell viability of human colorectal cancer lines after GLV-1h68 infection.** Cells were infected in culture at MOIs of 0.1 (**A**) and 1.0 (**B**) and assayed for viable cells using the MTT assay over a time course of 72 hours. Viability was measured in two sets of triplicates and averaged. Averages were normalized against the uninfected controls at each time-point which were considered to be 100% viable.

Cell viability assays were performed to assess the cytotoxicity of GLV-1h68 infection in culture at MOIs of 0.1 and 1.0 (Figure 2). While differences were observed between the cell lines and at different MOIs, all five human colorectal cancer cell lines displayed significant cytotoxicity after infection. GLV-1h68 displayed greatest cytotoxicity in HCT-116 cells and was least cytotoxic in HCT-15. At 48 hours post infection, only 19.2 ± 0.7% (MOI 0.1) and 1.8 ± 0.8% (MOI 1.0) of HCT-116 remained viable.

**Figure 3:**
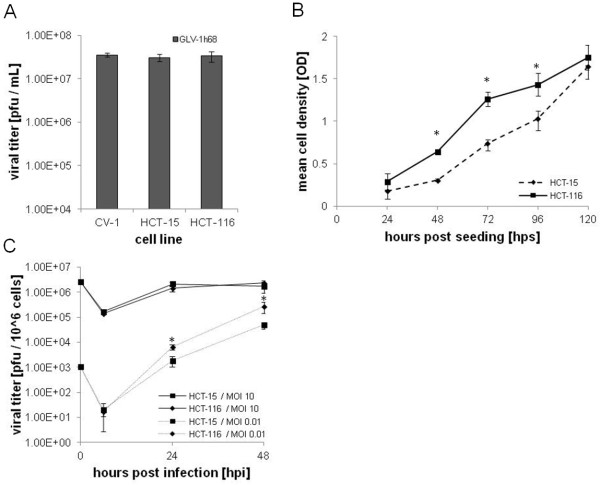
**Analysis of cell susceptibility to GLV-1h68 cytotoxicity in HCT-15 and HCT-116 CRC cells. A**) Plaque forming ability in CV-1 cells (standard), HCT-15 (low cytotoxicity), and HCT-116 (high cytotoxicity). All cell lines were tested in triplicates with a dose of 1x10^8^ pfu of GLV-1h68 for 24 hours at 37°C. Cells were then harvested and plaque forming ability was determined by standard plaque assay. Average titers were determined from triplicates and expressed as pfu/mL. Averages plus standard deviations were plotted. **B**) Cell proliferation of HCT-15 and HCT-116. Cells were seeded at a concentration of 1x10^3^ cells/well and cell proliferation was followed for 24, 48, 72, 96 and 120 hours. Cell lines were tested in quadruplicates and averages plus standard deviations are plotted. **C**) Replication of GLV-1h68 at low and high MOIs. Cells were infected at an MOI of 0.01 (low) or 10 (high) and samples were collected at 6, 24, and 48 hours after infection. Average titers were determined in from triplicate and normalized to pfu/1x10^6^ cells. Averages plus standard deviations are plotted.

We investigated whether the susceptibility to cytotoxicity by GLV-1h68 observed in the CRC cell lines might be due to factors affecting cell infectivity or viral spreading. Plaque formation in HCT-15 (low susceptibility) and HCT-116 (high susceptibility) was compared to CV-1 cells (our standard laboratory cell line for propagation of vaccinia virus). No significant difference in plaque number was observed after infection for 24 hours with the same number of pfu (Figure 3A). Evaluation of the growth in culture of HCT-15 and HCT-116 cells showed that HCT-116 cells proliferated significantly (P≤0.05) faster than HCT-15 cells during the first 72 hours (Figure 3B). After 24 and 48 hours of infection at an MOI of 0.01, mimicking the virus:tumor cell ratio *in vivo,* GLV-1h68 replicated significantly faster (P≤0.005) in HCT-116 cells compared to HCT-15 cells, although there was no significant difference at an MOI of 10 (Figure 3C).

To investigate whether antiviral type I IFN-α/β signaling in infected host cells contributed to virus susceptibility, HCT-15 and HCT-116 were infected with low dose (MOI 0.01) and high dose (MOI 5.0) of GLV-1h68. The supernatant of the infected cells was then analyzed by ELISA at different time points after infection (30, 60, 120, and 360 min, 24 h) for IFN-α and IFN-β secretion. Neither HCT-15 nor HCT-116 cells yielded measurable amounts of secreted IFN-α or IFN-β after infection with GLV-1h68 (data not shown). Therefore, the greater observed cytotoxicity of the CRC cell lines after GLV-1h68 infection was associated with greater efficiency of GLV-1h68 replication and greater proliferation rate of the cells, not with differences in infectivity or with the production of type I IFN.

### Analysis of virus-mediated marker protein expression

**Figure 4:**
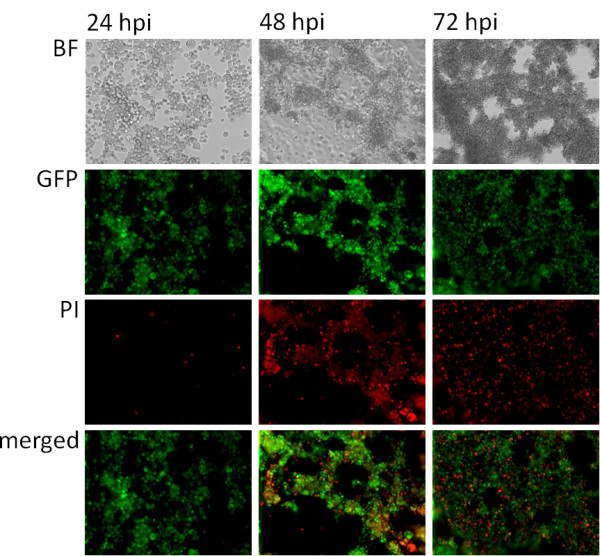
**Fluorescence microscopy of virus-mediated *Ruc*-GFP expression in HCT-116.** HCT-116 cells were infected at an MOI of 1.0 and monitored for 72 hours. (BF) shows bright field microscopic images of the morphology of virus-infected cells. (GFP) expression in infected cells was visualized by direct fluorescence microscopy; propidium iodide (PI) was used as a marker for dead cells. Colocalization of (GFP) and (PI) signal is shown in the (merged) images. All pictures were taken at 40x magnification.

The correlation of viral replication and cell death in GLV-1h68-infected CRCs was evaluated microscopically by identifying cells expressing the GLV-1h68-mediated *Ruc*-GFP transgene product in HCT-116 cells at two different MOIs. Cell morphology changes were followed in bright field images over the course of 72 h (Figure 4, upper row). Green fluorescent protein expression, as a marker for viral infection and replication, was visualized by fluorescence microscopy (Figure 3, second row). Propidium iodide staining was used to identify dead cells (Figure 3, third row). Infection, replication and gene expression of *Ruc*-GFP from GLV-1h68 occurred efficiently (Figure 4 & Additional file 1) in a MOI- and time-dependent manner after infection of all tested cell lines (data not shown). Cell death and CPE (cytopathic effects) increased following *Ruc*-GFP expression. The overlay image in Figure 4, third row, demonstrates the predominant coincidence of *Ruc*-GFP expression and propidium iodide staining in the same cells. Thus, GLV-1h68 infection and replication proceeded to cell death in a majority of cells imaged.

**Figure 5:**
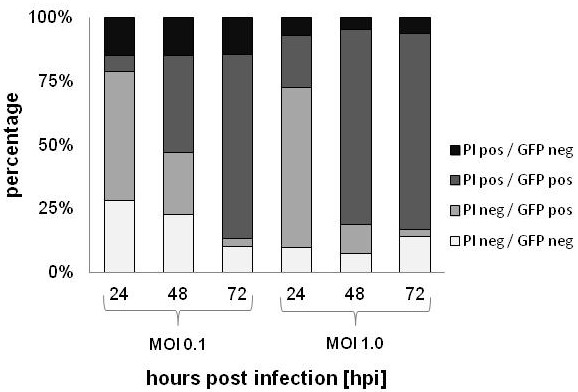
**Flow cytometry analysis of GLV-1h68-infected HCT-116 cells.** Cells were infected at MOIs of 0.1 or 1.0. Data represents the average distribution of uninfected/infected [GFP neg/pos] and viable/dead [PI neg/pos] cells over the course of 72 hours post infection.

This coincidence was also confirmed and quantified by flow cytometry analysis of GLV-1h68-infected (GFP^pos^) and dead cells (propidium iodide staining). After infection of HCT-116 cells with GLV-1h68 at an MOI of 0.1, the GFP^pos^ cell fraction increased from 57.1 ± 5.9% at 24 hours to 62.3 ± 3.5% at 48 hours and 75.4 ± 3.1% at 72 hours, consistent with the increased infection and replication of the virus. The fraction of these cells (GFP^pos^) that were also propidium iodide positive progressively increased from 6.4 ± 1.9% at 24 hours to 37.9 ± 1.8% at 48 hours and 72.2 ± 2.7% at 72 hours. At the last time point, the majority of cells were either virus-infected or dead (75.4% ± 3.1%). At an MOI of 1.0, the observed results were similar, except accelerated. In agreement with the *in vitro* cell culture results, GLV-1h68 showed highest efficacy of replication and cytotoxicity in HCT-116 and Colo 205, followed by HT-29 and SW-620 and was least effective in HCT-15 cells. Thus, GLV-1h68 efficiently infected, replicated in and ultimately killed human colorectal cancer cells in culture, as confirmed by FACS analysis (Figure 5 and Additional file 2).

### A single dose of GLV-1h68 causes tumor growth inhibition in HCT-116 and SW-620 xenografts

**Figure 6:**
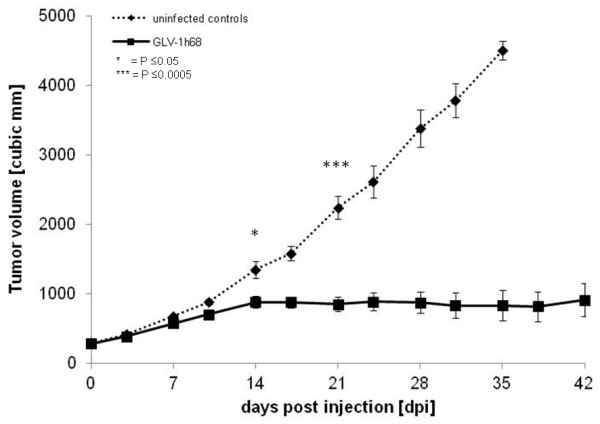
**Effects of a single administration of GLV-1h68 on tumor growth of HCT-116 tumor-bearing mice.** HCT-116 cells were implanted subcutaneously in the right hind leg of athymic nude mice and GLV-1h68 treatment was evaluated versus PBS treatment. Average tumor volume (ATV) for GLV-1h68-treated (n=10) and for PBS treated control mice (n=5) is plotted over time after treatment and one-way analysis of variance (ANOVA) was used to compare the data. P ≤ 0.05 was considered statistically significant; * = P ≤ 0.05, *** P ≤ 0.0005.

We next examined the efficacy of GLV-1h68 to target and affect the growth of HCT-116 (Duke’s type A) xenograft tumors in mice. HCT-116-tumor bearing mice were injected subcutaneously either with 5×10^6^ of GLV-1h68 (n=10) or PBS only (n=5) when the tumor volume reached an average of 250 mm^3^. Tumor volume was determined by physical measurement of the palpable tumors. Starting at 14 days post injection, animals receiving a single dose of GLV-1h68 had significantly (P≤0.05) inhibited tumor growth. This tumor growth inhibition became highly statistically significant (P≤0.0005) 21 days after virus injection and persisted from Day 28 through the end of the study at Day 42 (Figure 6). Overall, after injection of GLV-1h68, the tumors of virus-treated animals grew to three times their starting volume whereas tumors of untreated animals grew up to fifteen times their starting volume.

The general health of virus-treated animals was not adversely affected as indicated by only a slight decrease in net body weightof the tumor-bearing animals. Seventy percent (70%) of GLV-1h68-treated animals survived the duration of the study to Day 42, whereas all untreated animals had to be sacrificed due to high tumor burden by Day 35 (Additional file 3). GLV-1h68-treatment was evaluated in a second tumor xenograft model in mice to examine whether cell line- or stage-specific differences occurred using the slow responding cell line SW-620 (Duke’s type C). SW-620-tumor bearing mice were injected either with 5×10^6^ pfu GLV-1h68 (n=10) or PBS. Again, inhibition of tumor growth after a single injection of GLV-1h68 was observed, becoming statistically significant (P≤0.05) after 21 days after virus injection. Untreated tumors grew approximately two-fold bigger than virus-treated tumors after 35 days of treatment (Additional file 4). The results indicate that in this model, GLV-1h68 treatment effectively inhibited the growth of both CRC tumors, from HCT-116 (Duke’s type A, fast proliferating, highly susceptible to GLV-1 h68 replication and cytotoxicity in culture) and SW-620 (Duke’s type C, slow proliferating, less susceptible to GLV-1h68 replication and cytotoxicity in culture than HCT-116).

### Replication of GLV-1h68 after injection is confined to the primary tumor site

To analyze the specific tumor-targeting ability of GLV-1h68, animals bearing HCT-116 tumors (n=5) were sacrificed 3 and 14 days after intravenous injection of the virus. Various body organs (testes, spleen, liver, kidney, lungs) and the primary tumor were excised, prepared and analyzed by standard plaque assay to evaluate viral spreading in the animals (Table 1). At three days after injection, 2.94×10^5^ ± 5.54×10^5^ viral pfu were detected in the primary tumors, whereas negligible or no infective virus was detected in the body organs. Even well-vascularized tissues like the liver or lungs showed no or very low numbers of plaque forming vaccinia virus particles, indicating rapid and efficient clearance from the blood stream. Between three and 14 days post injection, a two-log increase in viral titers in the primary tumor tissue was detected whereas no virus (testes, spleen, kidneys) or negligible amounts of virus (liver, lungs) were found in normal tissues.

**Table 1:** Viral titers in mouse tissues or tumors

**Viral Titers (mean ± standard deviation)[pfu/g]**				
**Tissue**	**3 dpi**	**#**	**14 dpi**	**#**
**tumor**	2.94 × 10^5^ ± 5.54 × 10^5^	5/5	4.21 × 10^7^ ± 2.48 × 10^7^	5/5
**lungs**	4.21 ± 6.11	1/5	2.62 ± 3.59	2/5
**liver**	n.d.	0/5	3.88 ± 8.68	1/5
**spleen**	n.d.	0/5	n.d.	0/5
**kidney**	n.d.	0/5	n.d.	0/5
**testes**	n.d.	0/5	n.d.	0/5

The viral titers in mouse tissues or tumors are shown. HCT-116-bearing mice [n=5] were sacrificed 3 days or 14 days [dpi] after i.v. injection of 5x10^6^ pfu GLV-1h68. Tissues and tumors were surgically excised, homogenized and viral titers were determined by standard plaque assay. Titers are displayed as means of all animals sacrificed per time point and in # column of the table indicates the number of positively tested animals out of all the tested animals in the group. n.d. = not detected.

**Figure 7:**
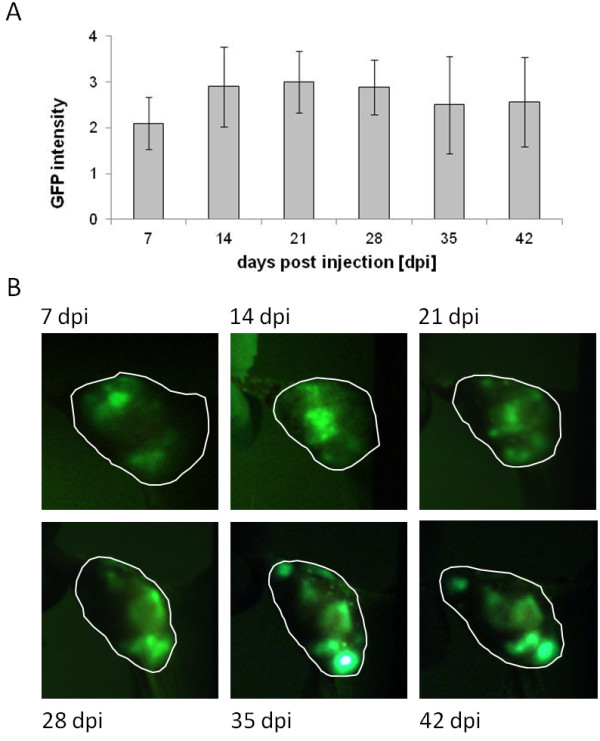
**Virus-mediated *Ruc*-GFP expression at the local tumor site of HCT-116-bearing mice. A**) Analysis of GFP fluorescence intensity in GLV-1h68-treated mice during the course of the experiment. GFP intensity is determined by using a four level visual scoring system; 0) no GFP signal, 1) one spot, 2) two or three local spots, 3) diffuse signal from half the tumor, 4) strong signal from whole tumor. Average scores with standard errors for groups of five mice at each time point are presented. **B**) Fluorescence imaging of GFP expression at the local tumor site is shown for one representative mouse.

**Figure 8:**
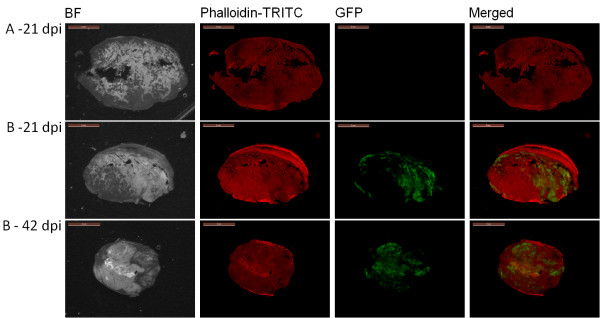
**Histochemical staining of HCT-116 tumor sections.** Microscopic images of histological sections of representative HCT-116 tumors from an untreated mous at 21 days post-injection [**A**] and GLV-1h68-treated mice [**B**] at 21 and 42 days post-injection [dpi] are shown. Images were obtained under bright field (BF) or fluorescence microscopy (Phalloidin-TRITC, GFP). Digital images were processed with GIMP2 (Freeware) and merged to yield pseudocolored images (merged). Whole tumor cross-sections (thickness = 100 μm) were labeled with Phalloidin-TRITC to detect *de novo* synthesis of actin as an indicator for live cells. GFP fluorescence indicates GLV-1h68 replication in the tumor.

GLV-1h68 infection and replication *in vivo* was also assessed by live, whole body fluorescence imaging and semi-quantitative measurement of fluorescence intensity in tumors and surrounding tissues, which was possible due to the negligible absorption of the GFP fluorescence signal by the tissues (Figure 6). GFP fluorescence in the tumor was clearly visible as early as 7 days post injection, increased until 21 days post injection, and remained relatively stable thereafter to Day 42 (Figure 7). The extent and intensity of GFP fluorescence in the tumor as a marker for GLV-1h68 infection and replication was also confirmed by histological examination of tumor sections at 21 and 42 days post injection. The merged images of Phalloidin-TRITC staining of actin and GFP fluorescence demonstrated that GLV-1h68 specifically infected cells within the tumor (Figure 8).

### Effects of a GLV-1h68 injection on the host immune system

To evaluate the role of the host immune system in virus clearance and the involvement in tumor growth inhibition, mice bearing HCT-116 tumors were either treated by a single intravenous injection of GLV-1h68 or treated by injection of PBS. Mice were sacrificed 21 days post injection, when the differences in tumor volume first became statistically significant (P≤0.0005). Tumor tissue lysates were prepared for immune-related antigen profiling. The data from virus-treated and untreated xenografts showed that treatment with GLV-1h68 was associated with increased levels of many pro-inflammatory cytokines and chemokines (GCP-2, KC/GRO alpha, IFN-γ, IP-10, IL-3, IL-6, Lymphotactin, M-CSF1, MIP-1 beta, MCP-1, MCP-3, MCP-5, RANTES) (Table 2). Only a few markers were down-regulated upon virus treatment (FGF-beta, MIP-1 alpha, MIP-1 gamma, SGOT).

**Table 2:** Comparison of mouseMAPS in homogenates of untreated or GLV-1h68-treated HCT-116 tumors

**Antigen**	**Untreated**	**GLV-1h68**	**P value**	**Ratio**
CD40	31.87 ± 10.5 fg/mg	193.41 ± 27.53 fg/mg	**	6.1
Eotaxin	1.24 ± 0.67 fg/mg	371.48 ± 122.26 fg/mg	*	300.0
GCP-2 mouse	0.03 ± 0.009 pg/mg	0.50 ± 0.29 pg/mg	*	19.3
KC/GRO	0.002 ± 0.001 pg/mg	0.073 ± 0.018 pg/mg	**	34.9
IFN gamma	1.61 ± 0.03 fg/mg	7.21 ± 0.35 fg/mg	***	4.5
IP-10	2.20 ± 0.71 fg/mg	145.28 ± 27.64 fg/mg	**	66.2
IL-1 alpha	14.18 ± 2.02 fg/mg	78.76 ± 7.82 fg/mg	***	5.6
IL-3	0.082 ±0.018 fg/mg	0.45 ± 0.18 fg/mg	*	5.5
IL-6	1.63 ± 0.57 fg/mg	20.24 ± 12.76 fg/mg		12.5
IL-10	9.89 ± 1.68 fg/mg	52.48 ± 0.46 fg/mg	***	5.3
IL-11	6.03 ± 1.70 fg/mg	41.71 ± 9.32 fg/mg	**	6.9
IL-17A	5.20 ± 0.93 fg/mg	52.96 ± 6.40 fg/mg	***	10.2
IL-18	1.41 ± 0.08 pg/mg	8.03 ± 2.33 pg/mg	*	5.7
Lymphotactin	14.25 ± 3.87 fg/mg	235.25 ± 36.29 fg/mg	***	16.5
M-CSF-1	318.57 ± 7.40 fg/mg	122.67 ± 35.91 fg/mg	**	38.5
MIP-1 beta	25.70 ± 8.90 fg/mg	1286.62 ± 206.46 fg/mg	***	50.1
MIP-2	1.20 ± 0.38 fg/mg	65.92 ± 30.24 fg/mg	*	55.1
MIP-3	0.065 ± 0.01 fg/mg	0.36 ± 0.03 fg/mg	***	5.5
MMP-9	9.93 ± 7.02 pg/mg	91.31 ± 60.77 pg/mg		9.2
MCP-1	14.55 ± 4.65 fg/mg	3332.49 ± 93.80 fg/mg	***	229.1
MCP-3	18.20 ± 7.64 fg/mg	2023.74 ± 386.90 fg/mg	***	111.2
MCP-5	11.91 ± 6.71 fg/mg	676.99 ± 187.73 fg/mg	**	56.8
RANTES	0.0052 ± 0.003 fg/mg	0.54 ± 0.11 fg/mg	**	105.7
TIMP-1 mouse	1.30 ± 0.22 pg/mg	12.22 ± 4.53 pg/mg	*	9.36
TNF alpha	5.38 ± 0.59 fg/mg	23.01 ± 2.51 fg/mg	***	4.27
FGF-b	107.94 ± 16.12 pg/mg	69.86 ± 15.26 pg/mg	*	1.5
MIP-1 alpha	0.23 ± 0.008 pg/mg	0.13 ± 0.034 pg/mg	*	1.7
MIP-1 gamma	0.94 ± 0.15 pg/mg	0.30 ± 0.03 pg/mg	**	3.1
SGOT	2.08 ± 0.72 pg/mg	1.08 ± 0.56 pg/mg		1.9

The table compares the levels of mouse immune-related protein antigen profiling in homogenates of untreated and GLV-1h68-treated HCT-116 tumors normalized to total protein per sample 21 days after virus injection [n=3]. The ratio of antigen in GLV-1h68-treated and untreated animals and the level of statistical significance are shown. * = P≤0.05, ** = P≤0.005, *** = P≤0.0005.

**Figure 9:**
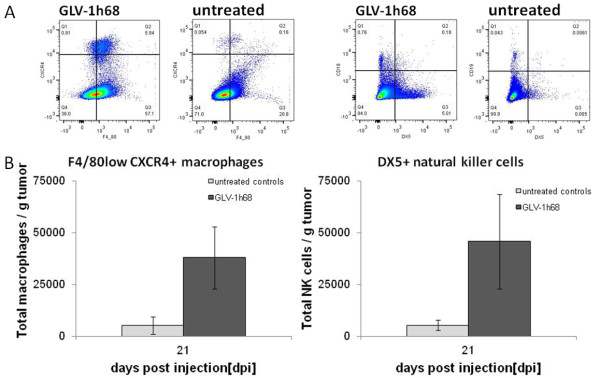
**Flow cytometry analysis of innate immune cells in untreated and GLV-1h68-treated tumors. A**) Representative flow cytometry plots of macrophage and NK cell populations in uninfected (n=4) and GLV-1h68-infected (n=5) HCT-116 tumors from flow cytometry analysis experiments at 21 days post treatment are shown. **B**) Quantification of total F4/80^low^ CXCR4^pos^ macrophages and CD19^pos^ DX5^pos^ NK cells per gram tumor after normalization by counting beads to quantify the total amount of cells per sample in untreated or GLV-1h68-treated HCT-116 tumors at 21 days post treatment. Average values with standard deviations are plotted. * = P≤0.05, ** = P≤0.005.

Immune cell infiltration in tumors was evaluated by preparing single cell suspensions from surgically excised tumors of untreated and treated animals 21 days post injection, and analysis by flow cytometry (Figure 9). Representing infliltrating innate immune cells, macrophages were identified using the cell antigen-specific markers F4/80 and CXCR4 and NK cells were identified using CD19 and DX5. HCT-116 tumors treated with GLV-1h68 had a significantly (P≤0.05) greater number of F4/80^low^ CXCR4^pos^ macrophages and CD19^pos^ DX5^pos^ NK cells than untreated tumors.

## Discussion

Colorectal cancer (CRC) is among the most abundant cancer types occurring in both sexes. Despite a generally good prognosis after treatment of early stage disease with standard therapy regimens, overall prognosis for patients diagnosed with stage IV colorectal cancer remains poor and recurrence rates after resection of primary tumors are high. Moreover, aggressive surgery and chemotherapy used to treat these cases are extremely debilitating on the patient. Therefore, the development of novel treatments for colorectal cancer that reduce recurrence rates and improve treatment efficacy especially of late stage CRC is highly desirable.

In these studies, we demonstrate for the first time that oncolytic vaccinia virus GLV-1h68 efficiently infected, replicated in, and lysed a variety of human CRC lines in culture. The observed oncolytic activity of GLV-1h68 occurred in both CRC cell types (colorectal adenocarcinoma and colorectal carcinoma) and appeared not to be dependent on the stage of disease, as the cell lines studied were derived from patients at all four Duke’s type clinical stages (from Duke’s type A, primary tumor, to Duke’s type D, metastatic disease). Viral replication, measured either by plaque formation or by expression of the virus-encoded *Ruc*-GFP, correlated with cell killing. Flow cytometry analysis and fluorescence microscopy confirmed that GFP expression was coincident with or preceded cell death in the virus-treated cell population. Our findings did show, however, that the different cell lines varied in degree of viral replication and cytotoxicity, and these differences were MOI-dependent. These results agree with reports by Ascierto *et al*. that GFP marker gene expression intensity upon infection with oncolytic vaccinia virus is cell line-dependent [29]. Yet, none of the five human colorectal cancer cell lines tested in culture showed any evidence of resistance to either infection, replication or subsequent lysis by GLV-1h68 in culture. These results suggest that GLV-1h68 oncolytic virotherapy could be generally applicable to the treatment of CRCs regardless of the cell type of origin and independent of the stage of disease, although their treatment could require different doses and/or regimens.

It is known that vaccinia virus depends on proliferating cells for its replication [30]. Thus, fast replicating tumor cells are better hosts for oncolytic vaccinia virus replication and consequently are more susceptible to the cytotoxic effects and subsequent oncolytic effects of GLV-1h68. Indeed, our results showed that the rate of replication of GLV-1h68 in the five CRC cell lines correlated with their rate of cell proliferation. Another factor contributing to the observed differences in the oncolytic effects of the different cell lines could be GLV-1h68’s ability to evade the host cell defense mechanisms (type-I IFN signaling, Toll-like receptor (TLR) signaling, interleukin signaling, apoptosis [31]). However, secretion of type-I IFNs which could have caused antiviral responses upon virus infection was not detected in the cell lines tested. This is consistent with Kirn *et al*[32] who reported that infection with oncolytic VACV directly blocks host cell production of type-I IFN. Certainly, a better understanding of the factors that affect vaccinia virus and host cell responses would be useful to improve the biological activity and thus the potential therapeutic efficacy of oncolytic virotherapy.

Our results also demonstrated that GLV-1h68 efficiently infiltrates, replicates in and inhibits the growth of human colorectal tumors in xenograft models. Tumor growth inhibition was coincident with the peak expression of *Renilla* luciferase-GFP in HCT-116 tumors 14 days post injection, suggesting that the invasion of GLV-1h68 and its replication in the tumor is the cause of the tumor growth inhibition. Furthermore, this result was achieved with only a single injection of GLV-1h68.

Viral infections trigger antiviral responses both in the infected cells (for example through type I IFN-α/β signaling) and in the infected organism (through cytokine and chemokine signaling). These responses recruit and activate components of the innate immune system to the site of infection to clear the host from the invading pathogens and protect it from a systemic infection. Therefore, the question remains whether the involvement of the host immune system is beneficial or detrimental to the virotherapy treatment. Many reports suggest an important role of the host immune system in eradication of tumors [33,34], yet antiviral tumor responses can limit viral replication and spread and therefore limit the effects of direct oncolysis [35]. As others have reported [12,20,23], we found that treatment of HCT-116 tumor-bearing mice with GLV-1h68 up-regulated (from at least 4-fold to 230-fold) pro-inflammatory cytokines and chemokines, like GPC-2, KC/GRO, IFN-γ, IP-10, IL-6, M-CSF-1 ,MIP-1 beta, MIP-2 and MIP-3, MCP-1, MCP-3, MCP-5, RANTES and TNF-γ. Interferon gamma is particularly important for immunity against intracellular pathogens, including vaccinia virus, and control of tumors and is produced predominantly by natural killer (NK) cells and natural killer T (NKT) cells as part of the innate immune response [36,37]. Additionally, a 66-fold increase was detected in IP-10 levels, a chemokine attracting T lymphocytes, monocytes and NK cells to the inflammation site [38]. Up-regulation of additional chemokines, like KC/GRO, GPC-2, GM-CSF 1, RANTES and MCP and MIP family members, also contribute to the recruitment of components of the innate immune system, exhibiting a similar role to IP-10. High levels of MCPs leading to chemotactic recruitment of macrophages, macrophage activation and subsequent release of MIPs at the site of inflammation after the treatment with GLV-1h68 should, and indeed, does promote a strong inflammatory response at the primary tumor site, as evidenced by the significant increase of infiltrating NK cells and macrophages in tumors of virus-treated animals.

It has been suggested that the tumor microenvironment presents a niche that promotes aberrant proliferation of malignant cells while offering protection from the host immune system. Cancers possess a broad repetoire of means to evade the host defense, including mimicking self, down-regulation of MHC molecules and interference with antigen presentation, production of factors that are lethal to or paralyzing T cells and lastly recruitment of so-called regulatory immune cells (tumor-infiltrating leukocytes, myeloid-derived suppressor cells (MDSCs) and regulatory T cells) that control effector immune cell functions and ultimately promote the evasion of immune surveillance [39].

We believe that infection of the tumor with GLV-1h68 disturbs this “immune-protected” microenvironment. Viral replication and direct oncolysis in the tumor leads to the observed increase in expression of chemoattractants and activators of maturation for components of the innate immune system (macrophages, NK cells, dendritic cells, neutrophils), thus creating a pro-inflammatory environment. Also, ongoing necrosis by viral oncolysis and the recruited components of innate immunity may facilitate influx of *de novo* immune cells into the previously immune-protected tumor microenvironment. Mature dendritic cells and macrophages might act as mediators that cross-talk with the naïve T cells to elicit an adaptive immune response against the tumor cells through activation by MHC class II tumor antigen presentation. Indeed, our results and observations demonstrate that, in contrast to untreated tumors, vaccinia virus infection triggers pro-inflammatory cytokine signaling in colorectal cancer tumors, followed by recruitment of cells of the innate immune system to the inflamed tumor site. These events could contribute to or augment the inhibition of growth and regression of the tumor, as proposed by Worschech *et al*[12].

Interestingly, we found SGOT levels in GLV-1h68-treated animals to be reduced compared to untreated animals. It has been shown that SGOT levels in the blood may be an indicator of liver or heart damage, or cancer. The elevated levels of SGOT in the untreated animals may simply reflect the higher tumor burden in these animals. Alternatively, the high levels of SGOT in the untreated animals could indicate the presence of liver metastasis, although no physical evidence of tumors were observed grossly.

For overall treatment efficacy, especially in late stage colorectal cancer, it is important that the oncolytic vaccinia virus GLV-1h68 also targets, invades and destroys distant metastases in addition to the primary tumor. In this study we demonstrated that GLV-1h68 infected and killed colorectal cancer cells derived both from primary tumors (HCT-116, Duke’s type A, HT-29, Duke’s type B and HCT-15, Duke’s type C) and metastatic sites (SW-620, Duke’s type C and Colo 205, Duke’s type D) in culture and *in vivo* (Table 3).

**Table 3:** **GLV-1h68 susceptibility in cell culture and *in vivo *of CRC cell lines derived from different clinical stages**

**Cell line**	**Clinical stage**	**GLV-1h68 replication in culture**	**GLV-1h68 cytotoxicity in culture**	**GLV-1h68 treatment in xenograft models**
HCT-116	Duke’s type A	fast	high	growth inhibition
HT-29	Duke’s type B	fast	high	non-responder
SW-620	Duke’s type C	slow	medium	growth inhibition
HCT-15	Duke’s type C	medium	low	non-responder
Colo 205	Duke’s type D	fast	high	n.d.

Comparison of five human CRC cell lines derived from patients at different clinical stage of disease regarding virus susceptibility (replication and cytotoxicity) in cell culture and treatment efficacy in mouse xenograft models of five human CRC cell lines at different clinical stages. n.d. = not determined.

Increasingly, reports have implicated colorectal cancer stem cells and their chemoresistant properties especially in the recurrence of tumors. Recently, Wang *et al*. reported that oncolytic vaccinia virus GLV-1h68 showed enhanced replication in human breast cancer stem-like cells in comparison to breast cancer cells [40]. These observations imply that conventional treatment approaches involving surgery and chemotherapy could significantly benefit in combination with treatment with oncolytic virus GLV-1h68 to target and eliminate remaining colorectal cancer (−initiating) cells and to reduce recurrence rates even in early stage detected CRCs and improve overall prognosis of the disease.

That GLV-1h68 is administered systemically and efficiently infects and kills tumors and metastases *in vivo*[20] presents a powerful tool for the non-invasive treatment of colorectal cancers, even in later stage progression of the disease when conventional treatments are ineffective.

## Conclusion

We have shown for the first time that the oncolytic replication-competent vaccinia virus strain GLV-1h68 can efficiently infect, replicate in, and lyse different colorectal cancer lines in culture, independent from their original stage of prognosis when obtained from the patient. Furthermore, we showed that GLV-1h68 specifically targets and exclusively replicates in HCT-116 (Duke’s type A) and SW-620 (Duke’s type C) colorectal tumor xenografts in mice. While surgical excision of primary colorectal tumors in combination with chemotherapy allows for a good survival prognosis but a recurrence rate of 40-60% of the disease, late stage colorectal cancer with metastasis to the lymph nodes or distant body organs is still fatal and treatment options palliative. The mild side effects of vaccinia virus administration and its ability to target and destroy colorectal cancer cells irrespective of the stage of disease makes oncolytic virotherapy, possibly in combination with conventional therapy, particularly promising for the future treatment and control of colorectal cancer.

## Abbreviations

ANOVA: Analysis of variation;ATV: Average tumor volume;BF: Bright field;CPE: *Cytopathic effect*;CRC: Colorectal cancer;dpi: Days post injection;FBS: Fetal bovine serum;FGF: *Fibroblast growth factor*;GFP: Green fluorescent protein;GM-CSF: *Granulocyte-macrophage colony-stimulating factor*;GCP: *Granulocyte chemotactic protein*;hpi: Hours post infection;IFN-α/β/γ: *Interferon alpha/beta/gamma*;IL: *Interleukin*;IP-10: *Interferon gamma-induced protein 10*;i.v.: Intravenous;KC/GRO: *Growth-regulated alpha protein*;LSD: Least significant difference;MAP: Multi analyte profile;M-CSF: *Macrophage colony-stimulating factor*;MIP: *Macrophage inflammatory protein*;MCP: *Monocyte chemotactic protein*;MDSCs: *Myeloid-derived suppressor*;MOI: Multiplicity of infection;NK: Natural killer;pfu: Plaque forming unit;PI: Propidium iodide;RANTES: *Regulated and normal T cell expressed and secreted*;RBC: *Red blood cell* Ruc: *Renilla* luciferase;rVACV: Recombinant vaccinia virus;s.c.: Subcutaneous;SGOT: *Serum glutamic oxaloacetic transaminase*;TLR: *Toll-like receptor*;TNF-α: *Tumor necrosis factor alpha*

## Competing interests

The research was supported by the Research and Development Division of Genelux Corp., San Diego, USA, and a Service Grant to the University of Würzburg, Germany also funded by Genelux Corp., San Diego, USA. NGC, JS, QZ, and AAS are employees and shareholders of Genelux Corporation. MOK is an employee of Genelux Corporation No competing interests exist for KE and LB. KE and LB are supported by graduate stipends from the University of Würzburg. The funders had no role in study design, data collection and analysis or decision to publish.

## Authors’ contributions

KE conceived the study, designed, performed and analyzed all experiments and wrote the manuscript. MOK participated in designing and analysis of flow analysis experiments. NGC participated in conceiving the study and drafting of the manuscript. LB participated in drafting the manuscript. JS participated in performing animal experiments and drafting the manuscript. QZ provided essential material and helped with statistical analysis of animal experiments. AAS participated in designing the study, writing the manuscript, and gave final approval of submission. All authors read and approved the final version of the manuscript.

## Authors’ information

NGC, JS, QZ and AAS are employees and shareholders of Genelux Corporation. MOK is an employee of Genelux Corporation. KE and LB are supported by graduate stipends from University of Würzburg. The funders had no role in study design, data collection and analysis or decision to publish.

## Supplementary Material

Additional file 1 **Fluorescence microscopy of virus-mediated *Ruc-*GFP expression in HCT-116.** CRC cells were infected with at MOI of 0.1 and monitored for 72 h. (BF) shows bright field microscopic images of the morphology of virus-infected cells. (GFP) expression in infected cells was visualized by direct fluorescence; propidium iodide (PI) was used as a marker for dead cells. Colocalization of (GFP) and (PI) signal is shown in the (merged) images. All pictures were taken at 40x magnification. Click here for file

Additional file 2 **Flow cytometry analysis of infection of various colorectal cancer cell lines.** Cells were infected at MOIs of 0.1 and 1.0. Data represents the average distribution of uninfected/infected [GFP neg/pos] and viable/dead [PI neg/pos] cells in triplicate over the course of 72 hours post-infection. Click here for file

Additional file 3 **Changes in net body weight and overall survival of untreated and treated animals.** A) Net body weight (g) was calculated using the following formula: body weight (g) – (tumor volume/1000 mm^3^). B) Overall survival is plotted as a Kaplan-Meier survival diagram; n=10 for GLV-1h68-treated group, n=5 for PBS-treated group. Click here for file

Additional file 4 **Effects of a single administration of GLV-1h68 on tumor development of SW-620 tumor-bearing mice.** SW-620 cells were implanted subcutaneously in the right hind leg of athymic nude mice and treated by injection GLV-1h68 or PBS. Virus treatment was tested versus PBS treatment. Average tumor volume (ATV) of [n=10 for GLV-1h68-treated, n=5 for PBS treated controls] mice is plotted and one-way analysis of variance (ANOVA) was used to compare the data. P ≤ 0.05 was considered statistically significant; * = P ≤ 0.05. Click here for file
