# Supplementary figures and images for: Growth inhibition of different human colorectal cancer xenografts after a single intravenous injection of oncolytic vaccinia virus GLV-1h68
Source: J Transl Med. 2013 Mar 26;11:79. doi: 10.1186/1479-5876-11-79 (PMC3621142; doi:10.1186/1479-5876-11-79)

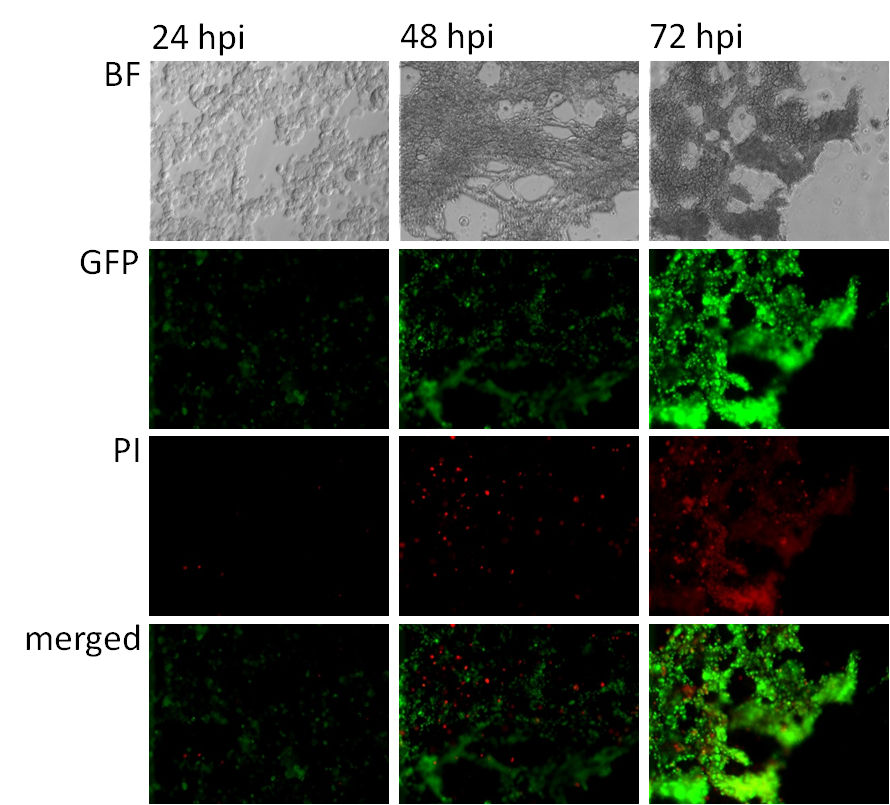

Supplement: Additional file 1 — Fluorescence microscopy of virus-mediated Ruc-GFP expression in HCT-116. CRC cells were infected with at MOI of 0.1 and monitored for 72 h. (BF) shows bright field microscopic images of the morphology of virus-infected cells. (GFP) expression in infected cells was visualized by direct fluorescence; propidium iodide (PI) was used as a marker for dead cells. Colocalization of (GFP) and (PI) signal is shown in the (merged) images. All pictures were taken at 40x magnification. [file 1479-5876-11-79-S1.tiff]

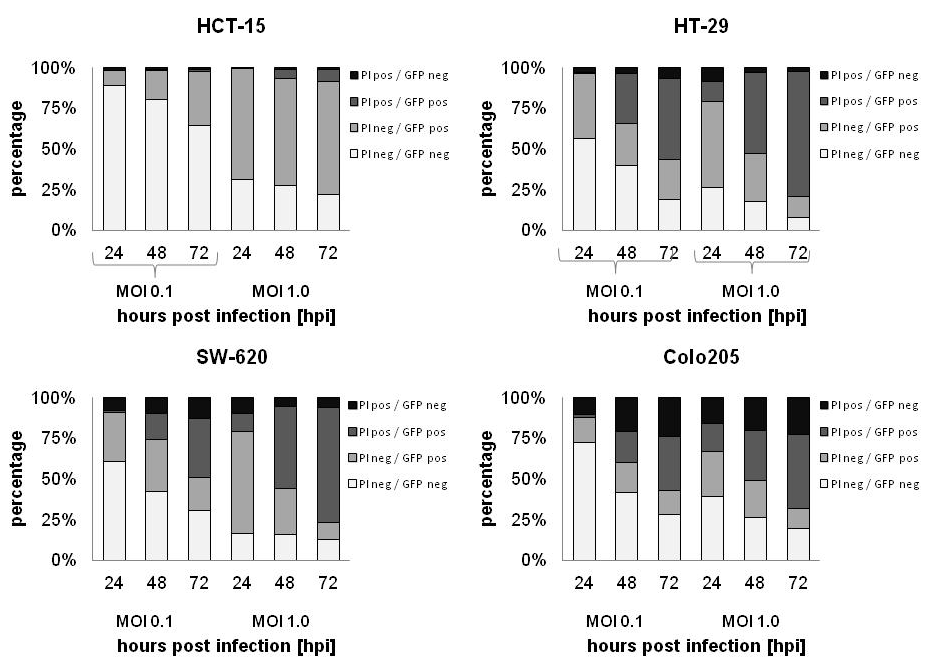

Supplement: Additional file 2 — Flow cytometry analysis of infection of various colorectal cancer cell lines. Cells were infected at MOIs of 0.1 and 1.0. Data represents the average distribution of uninfected/infected [GFP neg/pos] and viable/dead [PI neg/pos] cells in triplicate over the course of 72 hours post-infection. [file 1479-5876-11-79-S2.tiff]

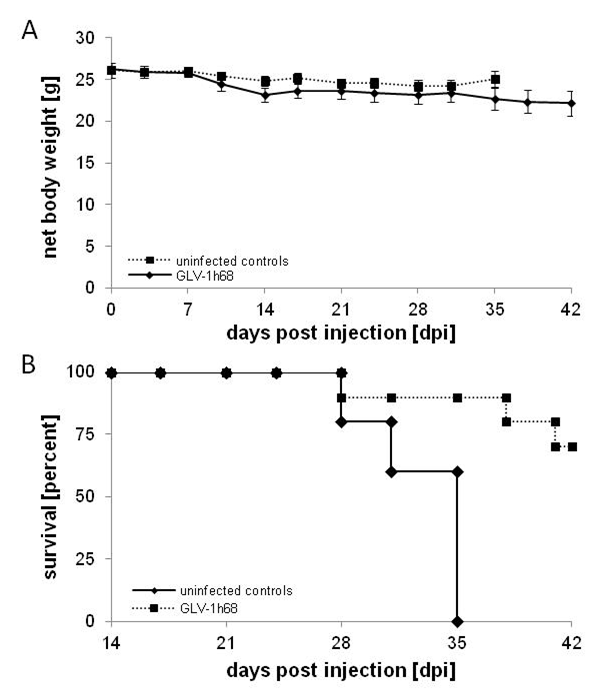

Supplement: Additional file 3 — Changes in net body weight and overall survival of untreated and treated animals. A) Net body weight (g) was calculated using the following formula: body weight (g) – (tumor volume/1000 mm3). B) Overall survival is plotted as a Kaplan-Meier survival diagram; n=10 for GLV-1h68-treated group, n=5 for PBS-treated group. [file 1479-5876-11-79-S3.tiff]

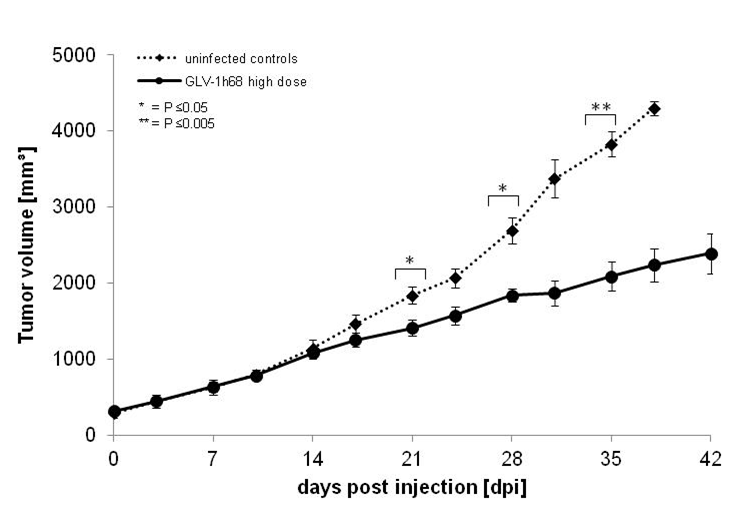

Supplement: Additional file 4 — Effects of a single administration of GLV-1h68 on tumor development of SW-620 tumor-bearing mice. SW-620 cells were implanted subcutaneously in the right hind leg of athymic nude mice and treated by injection GLV-1h68 or PBS. Virus treatment was tested versus PBS treatment. Average tumor volume (ATV) of [n=10 for GLV-1h68-treated, n=5 for PBS treated controls] mice is plotted and one-way analysis of variance (ANOVA) was used to compare the data. P ≤ 0.05 was considered statistically significant; * = P ≤ 0.05. [file 1479-5876-11-79-S4.tiff]
